# Parenthood and Women’s Subjective Well-being in a Low-income, High-fertility Context: A Case Study from Rural Gaza Province, Mozambique

**DOI:** 10.1007/s11113-025-09978-8

**Published:** 2025-10-31

**Authors:** Sarah R. Hayford, Luca Badolato, Victor Agadjanian

**Affiliations:** 1 Ohio State University, Ohio Columbus, US; 2 University of California Los Angeles, California Los Angeles, US

**Keywords:** Parenthood, Subjective well-being, Sub-Saharan africa

## Abstract

In rural high-fertility settings where people depend on subsistence agriculture, children are expected to provide material support to their parents in later life, with implications for physical health and material well-being of parents. Substantial research has examined these material consequences. Fewer studies have examined the implications of parenthood for subjective well-being in these contexts, in contrast to a larger body of research in low-fertility contexts. The existing studies of parenthood and subjective well-being in high-fertility contexts suggest that this relationship depends on parents’ gender and age, but do not distinguish between the impact of parent life stage and the impact of child age and other child characteristics. In this study, we draw on data from a population-based survey of ever-married women in rural Gaza Province, Mozambique, to show how mid-life women’s subjective well-being, measured as life satisfaction, is related to the number, age, and residential status of children. We also investigate whether the association between children’s characteristics and mother’s life satisfaction is mediated by other domains related to anticipated returns to childbearing, such as household economic conditions and mother’s physical and mental health. Results show that having young children in the household is negatively associated with life satisfaction, while having older children living outside the country is positively associated with life satisfaction. These associations are not fully explained by potential mechanisms such as economic conditions. We reflect on the implications of these findings in a context of changing livelihood strategies.

**Supplementary Information:**

The online version contains supplementary material available at 10.1007/s11113-025-09978-8.

## Introduction

A substantial body of empirical research in low-fertility contexts examines the relationship between parenthood and subjective well-being, i.e., happiness, life satisfaction, or other assessments of how well life is going. (Following the literature, we use the umbrella term “subjective well-being” for all of these measures.) Even in contexts with low and declining birth rates, most people continue to want children, presumably because they anticipate that parenthood will be pleasurable or rewarding in some way (Kohler & Mencarini, 2016; Sobotka & Beaujouan, 2014). Research on parenthood and subjective well-being attempts to discern whether these expected returns materialize. Results show that parenthood is not necessarily associated with higher subjective well-being: The association varies significantly across contexts and across parents’ characteristics, including age, gender, and socioeconomic status (Aassve et al., 2012; Billari, 2009; Billari & Kohler, 2009; Kohler et al., 2005; Margolis & Myrskylä, 2011).

There have been fewer studies of the relationship between parenthood and subjective well-being in high-fertility contexts. In these contexts, often characterized by small-scale agricultural economies and a lack of formal social safety nets, children provide clear material benefits to parents, and these material benefits are at the core of theories of high fertility (Caldwell & Caldwell, 1987; Caldwell, Orubuloye, and Caldwell 1992). In particular, the late onset and slower pace of fertility decline across Sab-Saharan Africa (SSA) compared to other regions are believed to be largely attributable to material conditions that make large families beneficial to parents (Bongaarts, 2017; Casterline, 2017; Church et al., 2023; Mbacké, 2017; Shapiro & Gebreselassie, 2013). For this reason, research on high-fertility contexts has tended to focus on these material benefits of parenthood, rather than more subjective assessments. The few existing studies of parenthood and subjective well-being in high-fertility contexts have found that having young children is negatively associated with subjective well-being, while older parents benefit from having more children (Clark et al., 2020; Conzo et al., 2017). However, it is not clear whether these different associations are due to children’s age or parents’ age, since previous studies typically include only one of these measures, and the role of children’s life course transitions such as leaving the parental home has not been directly examined. In addition, the importance of children’s economic contributions – the presumed motivation for high fertility – relative to other kinds of returns to parenthood is not clear. Given rapid and ongoing changes in social and economic conditions in SSA, including educational expansion, urbanization, and climate change, it is important to understand how parents experience the challenges and rewards of raising children.

In this study, we examine the association between women’s life satisfaction and the number, age, and residential status of their children in a high-fertility, low-income context in rural Mozambique. We draw on data from a population-based survey of ever-married women (n = 1892) that focuses on mid-life women, who may have both young children who require substantial care and older children who can provide support. Results show that the overall number of children is not significantly associated with life satisfaction, but there are variations depending on children’s age and where they live. While having young children living at home is negatively associated with life satisfaction, having older children in the home is not significantly associated with life satisfaction. Women with older children who have left the home report higher levels of satisfaction, especially if their children live outside of Mozambique. These associations are not fully mediated by measures of economic conditions or physical or mental health.

## Parenthood and Subjective Well-Being

Research across a range of low-fertility contexts has investigated the relationship between parenthood (operationalized as parental status, number of children, or recent births) and subjective well-being. This research is motivated by contemporary theories of fertility that propose that people have children primarily for subjective rather than material rewards (Bachrach & Morgan, 2013; Johnson-Hanks et al., 2011; Schoen et al., 1997). According to these theories, parents benefit from relationships with their children as well as, potentially, from the social status and social ties associated with parenthood. When children are young, these benefits may be offset by high demands of childcare, resulting in small net gains to subjective well-being, or even negative influences. When children are older, they require less care, resulting in larger net gains in well-being for parents.

Existing research confirms these hypotheses to some extent, but with substantial variation across parental characteristics, parental life course stage, and social and institutional context. In Britain and Germany, Myrskylä and Margolis (2014) found that, on average, subjective well-being increases in anticipation of childbearing and during the first year of parenthood, especially for first births, but then returns to pre-birth levels. They also found that this association is moderated by parental age and education: It is positive among older and more educated parents and negative among younger and less educated parents. In Australia, Matysiak et al. (2016) found a negative effect of parenthood on subjective well-being among parents who perceive substantial work-family conflicts (especially mothers), and a short-term positive effect among those with low work-family conflicts. In Germany, Pollmann-Schult (2014) found that although parenthood was positively associated with subjective well-being, the association was offset by the financial and time cost of raising children. In Poland, Baranowska and Matysiak (2011) found a positive effect of having a first child on subjective well-being only for mothers and a null incremental effect for higher-order children. In Hungary, Radó (2020) found that first and second births have a positive short- and long-term effect on subjective well-being for mothers and a positive short-term effect for fathers. In Russia, Mikucka (2016) found that first and, even more, second births have a positive and long-term effect on subjective well-being for both mothers and fathers. In China, Qian and Knoester (2015) found that parenthood is positively associated with subjective well-being only among older parents with adult children.

Comparative research illuminates the role of socio-institutional contexts. Using data across European countries, Aassve et al. (2012) found that while fathers’ subjective well-being does not vary across welfare regimes, mothers report higher subjective well-being in Nordic countries. Differences between men and women across countries are partially explained by institutions that shape the experience of parenting, including labor market structures, work-family policy, childcare availability, and gender norms. Mothers report higher subjective well-being than women without children in countries where institutions make it easier to reconcile parenthood with employment (Aassve et al., 2015). Using data from 22 OECD countries, Glass et al. (2016) found that work-family policies such as paid parental leave, work flexibility, and paid sick and vacation leave strongly moderate the association between parenthood and well-being, with the United States showing the largest negative gap in well-being between parents and childless individuals. In one of the few studies to include both high-income and low-income countries, Margolis and Myrskylä (2011) found, using World Values Surveys data from 86 countries, that the number of children is on average negatively associated with subjective well-being among adults under age 40. This association is weaker in countries with high public support for families. The association between the number of children and well-being gradually turns positive after age 40.

Research on the benefits of parenthood in high-fertility contexts has generally focused on the material returns that children provide (with the exception of large comparative studies that include some high-fertility countries such as Margolis & Myrskylä, 2011, cited above). However, there is a growing recognition of the importance of children as a source of cultural meaning, social status, and emotional support (Priebe, 2020; Smith, 2001). At the same time, people’s own assessment of their well-being is drawing more attention as an indicator of economic and social development (Stiglitz, Fitoussi, and Durand 2018). For this reason, more studies in low-income countries are including measures of subjective well-being.

A few studies have used these measures to analyze the association between parenthood and subjective well-being in high-fertility settings. Similar to findings across low-fertility countries, the relationship between parenthood and subjective well-being varies by parent’s age and gender. Using UNICEF’s Multiple Indicator Cluster Survey data from 35 low- and middle-income countries and an instrumental variable approach, Priebe (2020) found that having a third child positively affects the subjective well-being of mothers, especially of older mothers. Among older adults, having more children is associated with higher levels of life satisfaction (Clark et al., 2020; Conzo et al., 2017; Margolis & Myrskylä, 2011). In rural Ethiopia, Conzo et al. (2017) found a positive effect of the number of children on subjective well-being only among older fathers, while in rural Malawi, Clark et al. (2020) found a positive effect only among older mothers. Recent births and young children are negatively associated with parents’ well-being, especially for mothers, in both contexts (Clark et al., 2020; Conzo et al., 2017).

These studies in high-fertility contexts distinguish between the short-term implications of recent births and the longer-term impact of parenthood for older adults. Comparisons across these two stages implicitly conflate differences according to parents’ life course stage and children’s life course stage by assuming that the differential returns to parenthood among older adults are due to the age of their children. However, childbearing takes place over many years in high-fertility contexts, especially in sub-Saharan Africa, where completed fertility is higher, the mean ages at first marriage and first birth are lower, and inter-birth intervals are longer than in other regions (Bongaarts et al., 2017; Casterline & Odden, 2016). At mid-life, therefore, women may have both young children who still require substantial care and older children who are contributing to household activities or who have left the household. Both the material and the socioemotional returns to parenting likely vary by children’s age and residential status, so it is important to directly measure variation in subjective well-being according to these characteristics.

Previous studies in high-fertility contexts have not directly tested the mechanisms through which parenthood might be associated with subjective well-being. Building on theories of fertility that suggest economic returns to parenthood are a motivator for having children, scholars argue that the relationship between parenthood and subjective well-being is mediated by economic conditions (Clark et al., 2020; Conzo et al., 2017). Physical and mental health may also play a role in the relationship between parenthood and subjective well-being. Both the economic costs and the physical and psychological demands of raising children might be particularly high when children are young, although young children might bring joy and positively influence mothers’ mental health. At the same time, financial and material support from adult children is a key mechanism linking fertility and subjective well-being, and having supportive adult children might also improve physical and mental health. Clark et al. (2020) found that recent births were not significantly associated with women’s mental health but that the number of children was positively associated with mental health among older women. Priebe (2020) found that having a third child was positively associated with satisfaction with friendships, family life, and treatment by others, which in turn might improve mental health. Stanca (2012), in a comparative study across 96 countries, found that the negative effect of parenthood on subjective well-being was largely explained by an indicator of financial satisfaction. In Thailand, a middle-income country without a formal pension system to support older adults, both financial conditions and physical health contribute to the positive association between parenthood and subjective well-being (Pimpawatin & Witvorapong, 2023). Further research is necessary to assess whether and to what extent economic conditions, physical health, and mental health mediate the association between parenthood and subjective well-being in high-fertility contexts.

## Setting

Our study is based on survey data collected in rural areas of Gaza Province, Mozambique, one of the poorest countries in the world (World Bank, 2023). As other rural areas in sub-Saharan Africa, this setting is characterized by early onset of childbearing and high fertility, with the total fertility rate in Gaza around 5.3 children per woman for the cohort of women in our study (Ministério da Saúde (MISAU), Instituto Nacional de Estatística (INE), and ICF International (ICFI) 2013). Rural Gaza’s economy is primarily characterized by rain-dependent subsistence agriculture, and farm work is mainly done by women. Male labor migration is common, especially to the Republic of South Africa (RSA), Mozambique’s much more developed neighbor (Crush & Frayne, 2010). Although employment abroad is expected to generate substantially higher financial returns than domestic employment, the returns to male labor migration have become increasingly complex and variable; the impact of men’s migration on the health and well-being of their non-migrating wives who remain in Mozambique varies depending on the economic and social experience of migration (Agadjanian & Chae, 2023; Agadjanian et al., 2021a, 2021b; Jansen & Agadjanian, 2024). Few women in this setting work in the formal labor market, but women frequently engage in small-scale production and sales to produce a limited income (Agadjanian et al., 2021a, 2021b). There is no formal system of unemployment insurance or social welfare payments. A modest social security system offers some financial support to poor older adults (age 60 +) whose households have no other sources of income (Branco & Andrés, 2019). In this context, as in other settings with limited formal social safety net programs, support from children may be particularly relevant for mothers’ well-being.

In this as in other contexts where people depend on subsistence agriculture, children make substantial contributions to household functioning from a young age, including household chores, supervision of younger children, and agricultural tasks (Aufseeser et al., 2018). In the study area, there are limited formal childcare facilities. Virtually all children enroll in school at some point; although there are no school fees for the 1st through 9th grades, families pay for uniforms and school supplies. Because of these costs, and because of the limited availability of secondary schools in the area, many students leave school without completing secondary education, and very few proceed to post-secondary education. In a survey of children carried out in one of the four districts of our study site in 2017, 97% of children age 14–17 had ever been enrolled in school, while only 75% were currently enrolled (authors’ calculations from survey data, Family Migration and Early Life Outcomes project)^1^. Marriage is a central aspect of transition to adulthood, and early marriage is common in the study site. According to data from the Demographic and Health Surveys, the median age at first marriage in Gaza Province is 19 for women and 23 for men for current cohorts of young adults (Instituto Nacional de Estatística (INE) and ICF 2024).

## Current Study

This study extends previous research on the association between parenthood and subjective well-being in high fertility contexts by assessing variation across children’s age and residential status. We analyze a sample of rural mid-life women, many of whom have both young children and older children and both coresident and non-coresident children. We directly compare associations with well-being for younger children and older children among women of similar ages, and we test for the role of economic conditions, physical health, and mental health in mediating these associations.

We operationalize subjective well-being using a measure of overall life satisfaction, described in detail in the methods section. Previous research has used a range of measures, including life satisfaction, quality of life, and happiness. Life satisfaction is typically measured either on a four- or five-point scale (e.g., Clark et al., 2020; Mikucka, 2016; Priebe, 2020). The Cantril ladder, a measure where the top represents “the best possible life for you,” is measured from 0–10 and is typically interpreted as life satisfaction or quality of life (e.g., Conzo et al., 2017; Pollmann-Schult, 2014). Happiness can be measured either on a four-point scale, as in the World Values Surveys (e.g., Margolis & Myrskylä, 2011; Stanca, 2012) and in other surveys (e.g., Baranowska & Matysiak, 2011), or on an 11-point scale (e.g., Aassve et al., 2012, 2015). There does not appear to be consistent variation in findings on the association between parenthood and subjective well-being depending on the measure used, and studies that have used both life satisfaction and happiness either in the main analyses or robustness checks have not found substantial differences in results for the two measures (Aassve et al., 2015; Myrskylä & Margolis, 2014; Priebe, 2020).

***Hypotheses***

Overall, we expect that children are positively associated with women’s subjective well-being, given the strong pronatalist norms and high social value accorded to children in this context and consistent with theories of high fertility (H1). However, we expect that this association varies by children’s age and residential status, as outlined below.

Because young children are more demanding in terms of parental care, we hypothesize that the association with mothers’ subjective well-being is more weakly positive or even negative for young children (H2). Older children require less care and are better able to provide support to mothers and households, so we hypothesize that positive associations with well-being are strongest for older children (H3).

Existing theory and empirical evidence suggest potentially competing hypotheses for the relationship between children’s residential status and mothers’ subjective well-being. On the one hand, children who are living in the household may be more likely to help with domestic and agricultural tasks, contributing to increased well-being for mothers (H4a). On the other hand, children who are living outside of the household may require less care from mothers, changing the nature of the relationship and improving well-being. In addition, children may provide financial support to mothers if they are engaged in income-generating work, especially in higher-earnings settings such as RSA. Thus, having children who live away from the household might be more positively associated with subjective well-being (H4b).

These hypotheses imply different pathways connecting parenthood and subjective well-being. If care for young children is physically and mentally demanding (H2), associations with well-being may be mediated by physical and mental health. If older children contribute to well-being primarily through economic support (H3, H4b), associations may be mediated by measures of economic conditions.

### Analytic approach

We assess cross-sectional associations between life satisfaction and the current number, age, and residential status of children. Existing research on childbearing and subjective well-being uses a variety of study designs and analytic approaches, including both cross-sectional (Aassve et al., 2012, 2015; Margolis & Myrskylä, 2011) and longitudinal designs (Baranowska & Matysiak, 2011; Myrskylä & Margolis, 2014); comparisons of parents vs. non-parents (Stanca, 2012) and analysis of the number of children (Clark et al., 2020; Conzo et al., 2017); and fixed-effects models or instrumental variables to account for selectivity (Cetre et al., 2016; Priebe, 2020). We base our analytic decisions on the goals of our analysis. Fixed-effects approaches are not well-suited to capturing the long-term implications of childbearing, since they require comparisons across time periods where other characteristics can be assumed to be stable. Because of this limitation, studies focusing on older adults typically use cross-sectional designs (Clark et al., 2020; Conzo et al., 2017). Following this model, we use a cross-sectional design to understand associations between subjective well-being and having older and younger children. Our study captures patterns and associations based on long-term life course decision-making rather than isolating short-term causal effects of a single transition. We discuss this issue further in the data and methods section.

## Data and Methods

We use data from the fifth wave of Men’s Migrations and Women’s Lives (MMWL) project, a longitudinal population-based survey conducted in four districts of rural Gaza Province, Mozambique. The first survey wave took place in 2006, with a sample of 1678 married women of reproductive age (18–40). MMWL used a two-stage sample design, with 56 villages (14 in each district) selected with probability proportional to size and 30 women randomly selected within each village. Additional full waves of data collection (with sample refreshment) were carried out in 2009 (Wave 2) and 2011 (Wave 3), with a shorter survey in 2014 (Wave 4) and another wave in 2017 (Wave 5). This analysis primarily draws on the Wave 5 data. (For a few cases with missing data on stable characteristics, we used responses from previous waves to fill in values.) Mortality in the sample was around 10% by Wave 5 (Agadjanian et al., 2021a, 2021b). Of the surviving women, 82% were re-interviewed in Wave 5, for a total sample size of 1892 women. About 6% of cases were missing data on predictor or outcome variables (n = 116, including five cases with missing values on life satisfaction). We imputed missing values on predictor variables using multiple imputation with chained equations (*mi impute* in Stata version 18) based on 20 imputed datasets.

Our outcome measure is overall life satisfaction measured at Wave 5. Women were asked, “Thinking of your life in general, are you very satisfied, rather satisfied, a little satisfied, or are you not satisfied with your life now?” Responses were coded 1–4, with 4 being very satisfied.

The main predictor is the woman’s number of living children at Wave 5, calculated by adding children reported living in the household and children age 15 and older reported living permanently outside of the household.^2^ Because it is rare for pre-adolescent children to live permanently away from the household, the survey did not ask about these children, and they are not included in the total. We further break down the total number of children by age and residence in order to account for variation in the association with life satisfaction according to children’s characteristics. Among coresident children, we distinguish pre-school-age children (age 0–5), school-age children (age 6–14), and adolescent and young adult children (age 15 and older). These age categories roughly reflect the degree of parental care and investment needed and the degree to which children might provide labor or other material support to parents; exploratory analyses did not find significant variation by age among children within each age category. We use age 15 as the cutoff for adolescent/young adult children, rather than the legal age of majority (18), because adolescent children in this context frequently take on meaningful responsibilities in the household. Among non-coresident children age 15 and older, we distinguish between those living elsewhere in Mozambique (including in the same community) and those living outside of Mozambique, mostly in RSA. In exploratory analyses, we tested for differences between sons and daughters in associations with mother’s subjective well-being and found few statistically significant differences. We therefore do not include child gender in our models. (Results from these models are included in an appendix, as noted below.)

We control for demographic and life history measures that might be related to both life satisfaction and the number of children: mother’s age, number of child deaths, and marital status and characteristics. (We do not interpret the coefficients for control variables in the results section; see Westreich & Greenland, 2013.) We model age as a quadratic function and child deaths as a linear function. (All child deaths reported over the reproductive years are included in the count; most of these child deaths were in the relatively distant past. Only nine women in the sample reported any child deaths in the three years preceding the survey.) Marital status and characteristics are measured as a four-category variable that incorporates husband’s migration experience, based on previous research using these data that shows a strong relationship between husband’s migration and women’s experiences, including health and fertility (Agadjanian & Chae, 2023; Agadjanian et al., 2011; Jansen & Agadjanian, 2024). The four categories are unmarried, married to a non-migrant (reference category), married to a “successful” migrant, and married to an “unsuccessful” migrant. Migrant success is a subjective assessment based on the woman’s response to a question about whether the household is better off since the husband migrated. It reflects both the migrant’s economic returns and the respondent’s perception of his commitment and responsibility to the family.

After estimating an initial set of models to describe the relationship between children’s age and coresidence and mother’s well-being, we estimate a second set of models including physical health, mental health, and household economic conditions as potential mediators. We use two measures of physical health, self-rated health (4-point scale, dichotomized as excellent/good vs. so-so/bad) and how often in the past month the respondent felt pain that limited daily activities (3-point scale, dichotomized as many times/sometimes vs. never). Mental health is captured by the frequency of feeling sadness/depression and worry/anxiety in the past month (both measured using 3-point scales, dichotomized as many times/sometimes vs. never). We incorporate both objective and subjective measures of household economic conditions. There are three objective measures: a 4-point scale rating household possession of durable goods; whether the household owns any cattle, a traditional marker of wealth in this rural context; and a measure of household food security. Food security is measured on a 3-point scale, where three is the most secure, calculated by averaging responses to six questions adapted from the Household Food Insecurity Scale (question text is included in Appendix 1). Our subjective measure of economic well-being is based on a question asking respondents to categorize their household relative to other nearby households: “In your opinion, are the living conditions of your household better, worse, or about the same as the majority of households in this community?” This measure is dichotomized as better vs. same/worse.

These measures could plausibly be considered confounders rather than mediators if they are also related to the reasons that women have more or fewer children. Physical health, mental health, and household economic conditions are measured at the time of the survey, while childbearing took place prior to the survey, which somewhat mitigates this concern, but current health and economic conditions may be correlated with conditions at previous time points before childbearing. Our primary goal in this analysis is to understand the nature of possible associations between childbearing biographies and later life outcomes; we present tests of statistical mediation as a way to shed light on these associations.

We treat the 4-category life satisfaction measure as a continuous variable and estimate ordinary least squares regression models, consistent with previous analyses of life satisfaction (e.g., Clark et al., 2020; Margolis & Myrskylä, 2011; Priebe, 2020; Stanca, 2012). In exploratory analysis, we tested ordered logit models and partial proportional odds models (Williams, 2006, 2016) and found that results were largely consistent in terms of statistical significance, direction, and relative magnitude of associations. We prefer the OLS models for ease of interpretation; results from other specifications are included in Appendix 2. To formally assess mediation of the association between number of children and subjective well-being, we use seemingly unrelated estimation (*suest* in Stata version 18) to test for differences between coefficients in nested models (Mize, Doan, and Long 2019).

As part of our exploratory analyses, we conducted multiple robustness tests to better understand the relationship between the number, age, residential status, and gender of children and women’s subjective well-being. In addition to the preferred models presented in Tables 1 and 2 in the results section, we tested (1) models with measures of each age and residential category included separately, rather than in the same model, to test the independence of associations (Table A2.2); (2) models using dichotomous measures of whether women had any children in each age and residential category, rather than continuous measures of the number of children in each category (Table A2.3); (3) models with interactions between the number of children in each age and residential category to test for moderation (Table A2.4); (4) models testing the number of sons and daughters in each age and residential category separately (Table A2.5). As noted in the preceding paragraph, we also compared different functional forms for the dependent variable (Table A2.1). Results from these additional specifications are included in Appendix 2. 

## Results

Table 1 shows the distribution of overall life satisfaction, children’s age and characteristics, and other individual and household characteristics in the sample. On a scale of 1–4, where 4 is very satisfied, the average level of satisfaction is 2.7. Women have an average of 4.4 children, with a range of 0 to 12. The largest proportion of children fall into the category of school age children (6–14), with an average of just under two children per woman in this age range. Both young children and older children are also common in the sample; women have an average of 0.7 children age 0 to 5 and 1.1 children age 15 and up in the household. Of children living outside of the household, most (87%) are in Mozambique.

**Table 1:** Distribution of dependent and independent variables

	Mean (sd) or %	Range	N
Overall life satisfaction	2.7 (0.8)	1–4	1887
Number of living children^a^	4.4 (1.9)	0–12	1892
Number of children:			1892
Age 0–5 in household	0.7 (0.8)	0–4	
Age 6–14 in household	1.9 (1.2)	0–5	
Age 15 and older in household	1.1 (1.2)	0–6	
Away from household, living in Mozambique	0.5 (0.8)	0–5	
Living outside of Mozambique	0.1 (0.3)	0–3	
Age	39 (6.3)	25–60	1874
Marital status and characteristics			1892
Unmarried	21%	–	
Married to non-migrant	61%	–	
Married to successful migrant	29%	–	
Married to unsuccessful migrant	10%	–	
Number of child deaths	0.8 (1.1)	0–8	1892
Self-rated health (good/excellent vs. so-so/bad)	78%	–	1892
Physical pain in past month (sometimes/many times vs. never)	35%	–	1892
Sadness/depression in past month (sometimes/many times vs. never)	64%	–	1892
Worry/anxiety in past month (sometimes/many times vs. never)	62%	–	1892
Household durable possessions	1.9 (1)	1–4	1795
Household owns cattle	34%	–	1795
Household food security	2.6 (0.4)	1–3	1795
Relative household wealth (better than most households vs. same or worse)	41%	–	1795

Data: Men’s Migrations and Women’s Lives project. N = 1892 women interviewed at W5. Distribution of non-missing values. ^a^Excludes children under age 15 living permanently away from the household

The average age of women in the sample is 39 years old. Only 21% of women are unmarried. Those who are married are approximately evenly divided between women married to a non-migrant (40%) and women married to a migrant. Among women married to migrants, about three quarters think that their household is better off since the migrant left (“successful” migrant). (Specifically, 73% of women married to migrants report that their household is better off: 29%/(29% + 10%) = 73%). About 78% of women report good or excellent health, although about one third report pain that disrupted daily activities at least sometimes in the past month. Both depression and anxiety are fairly common in this sample, with about two thirds of women reporting feeling depressed and similar levels reporting feeling anxious at least sometimes in the past month. For economic well-being, on a four-point scale of household durable goods, the average score was 1.9, and about one third of women lived in households that owned cattle. The average level of food security was 2.6 (where 3 is the most secure). About 41% of women felt that their household was better off than others in their community.

Table 2 shows results from OLS models predicting life satisfaction based on number and characteristics of children and demographic and life course controls. Overall, the number of living children is not significantly associated with life satisfaction (Model 1). Thus, Hypothesis 1 is not supported. In Model 2, children are categorized by age and coresidential status. Consistent with Hypothesis 2, the number of young children (under age 6) is negatively associated with life satisfaction. The numbers of school age and adolescent/young adult children living in the household are not significantly associated with mother’s life satisfaction, and thus Hypotheses 3 and 4a are not supported. Looking at children living outside of the household, the number of children living outside of Mozambique (primarily in RSA) is positively associated with life satisfaction, providing some support for Hypothesis 4b. In particular, for each additional child living outside of Mozambique, women report a level of life satisfaction about 0.15 units higher on a four-point scale.

In supplementary analyses, we tested models including each age and residence category one-by-one (see Appendix 2, Table A2.2). Coefficients in these models were nearly identical to those in Model 2, suggesting that children in different age and residence categories have largely independent associations with life satisfaction. We also tested models with interactions and found no statistically significant moderation – having older children in the household, for example, does not moderate the negative association of having young children in the household with life satisfaction (Table A2.7). Finally, we tested models distinguishing between boys and girls in each age and residential category (Table A2.5). In these models, girls living outside the household in Mozambique are positively associated with women’s life satisfaction, while boys living outside the household in Mozambique are not significantly associated with life satisfaction, consistent with the preferred model in Table 2. In other age and residential categories, coefficients for boys and girls are not significantly different from each other.

Table 3 adds possible mechanisms connecting fertility and life satisfaction. Model 1 is a baseline model with the full specification for age and residence of children (replicating Table 2, Model 2). The subsequent models in Table 3 add physical health, mental health, and economic conditions as potential mediators, with Model 5, the full model, including all domains. The magnitudes of the coefficients for number of living children in different age and residence categories are lower in the models that include measures of economic conditions (Model 4, Model 5) relative to the baseline model (Model 1). The change in magnitude is largest for older children living outside of Mozambique (b = 0.10, Model 4; b = 0.11, Model 5 vs. b = 0.15, Model 1), and the coefficient does not reach conventional levels of statistical significance in Model 4 (p = .08). Seemingly unrelated estimation confirms that the differences in coefficient magnitude between Models 1 and 4 are statistically significant, suggesting that the association between children’s age/residential status and mother’s subjective well-being is at least partially mediated by economic conditions. Other potential mediators do not explain the relationship between the number of children and life satisfaction. In particular, neither worse physical health (Model 2) nor worse mental health (Model 3) contributes to the negative association between having young children at home and life satisfaction, based on comparison of coefficient magnitudes and confirmed by significance tests from seemingly unrelated estimation.

For the most part, physical and mental health and economic conditions are associated with life satisfaction as would be expected based on previous research. Surprisingly, reporting sadness or depression more often in the past month is positively associated with life satisfaction in the full model (Model 5), but this model includes several highly correlated constructs and coefficients should be interpreted with caution.

**Table 2:** OLS regression predicting life satisfaction as a function of number and characteristics of children

	Model 1			Model 2		
	b	se		b	se	
*Number and characteristics of children*						
Number of living children^a^	− 0.01	0.01				
Children living in the household						
Children 0–5 in the household				− 0.09	0.03	***
Children 6–14 in the household				− 0.03	0.02	
Children 15 and older in the household				0.02	0.02	
Children 15 and older living outside the household						
Children living in Mozambique				0.03	0.03	
Children living outside Mozambique				0.15	0.06	**
*Controls*						
Child deaths	− 0.004	0.02		− 0.003	0.02	
Age	− 0.04	0.03		− 0.05	0.03	
Age squared	0.001	0.0004		0.0005	0.0004	
Marital status (reference = married to non-migrant)						
Unmarried	− 0.36	0.05	***	− 0.38	0.05	***
Married to a “successful” migrant	0.22	0.05	***	0.23	0.04	***
Married to an “unsuccessful” migrant	− 0.18	0.06	**	− 0.18	0.06	**
Intercept	3.62	0.64	***	4.01	0.66	***

Data: Men’s Migrations and Women’s Lives project. N = 1887 women interviewed at W5, missing values on independent variables imputed using multiple imputation with chained equations. *: *p* < .05; **: *p* < .01; ***: *p* < 001. ^a^Excludes children under age 15 living permanently away from the household

**Table 3:** OLS regression predicting life satisfaction as a function of number and characteristics of children, accounting for potential mechanisms

	Model 1 Baseline			Model 2 Physical health			Model 3 Mental health			Model 4 Economic conditions			Model 5 Full model		
	b	se		b	se		b	se		b	se		b	se	
*Number and characteristics of children*															
Children 0–5 in the household	− 0.09	0.03	***	− 0.09	0.03	***	− 0.09	0.03	***	− 0.06	0.02	*	− 0.06	0.02	*
Children 6–14 in the household	− 0.03	0.02		− 0.03	0.02		− 0.03	0.02		− 0.02	0.02		− 0.02	0.02	
Children 15 + in the household	0.02	0.02		0.01	0.02		0.02	0.02		0.005	0.02		− 0.001	0.02	
Children living outside the household in Mozambique	0.03	0.03		0.04	0.02		0.04	0.03		0.02	0.02		0.03	0.02	
Children living outside Mozambique	0.15	0.06	**	0.15	0.06	**	0.17	0.06	**	0.10	0.06		0.11	0.06	*
*Physical health*															
Good self-rated health				0.44	0.04	***							0.33	0.04	***
Pain in the last month				− 0.08	0.04	*							0.01	0.04	
*Mental health*															
Sadness/depression in the last month							0.03	0.05					0.17	0.05	***
Worry/anxiety in the past month							− 0.15	0.05	**				− 0.11	0.04	*
*Household economic conditions*															
Household possessions scale										0.03	0.02		0.03	0.02	
Household owns cattle										0.1	0.04	**	0.09	0.04	*
Food security										0.51	0.05	***	0.48	0.05	***
Household relative economic status										0.2	0.04	***	0.18	0.04	***
*Controls*															
Child deaths	− 0.003	0.02		− 0.01	0.02		-0.0001	0.02		0.004	0.02		− 0.001	0.02	
Age	− 0.05	0.03		− 0.04	0.03		− 0.05	0.03		− 0.04	0.03		− 0.03	0.03	
Age squared	0.0005	0.0004		0.0004	0.0004		0.0005	0.0004		0.0004	0.0004		0.0003	0.0004	
Marital status (reference = married to non-migrant)															
Unmarried	-0.38	0.05	***	-0.36	0.05	***	− 0.37	0.05	***	− 0.27	0.05	***	− 0.26	0.05	***
Married to a “successful” migrant	0.23	0.04	***	0.18	0.04	***	0.24	0.04	***	0.12	0.04	**	0.09	0.04	*
Married to an “unsuccessful” migrant	− 0.18	0.06	**	− 0.15	0.06	*	− 0.18	0.06	**	− 0.05	0.06		− 0.04	0.06	
Intercept	4.01	0.66	***	3.43	0.64	***	4.12	0.66	***	2.13	0.64	***	1.76	0.63	**

Data: Men’s Migrations and Women’s Lives project. N = 1887 women interviewed at W5, missing values on independent variables imputed using multiple imputation with chained equations. *: *p* < .05; **: *p* < .01; ***: *p* < 001.

## Discussion and Conclusion

In low-income, high-fertility contexts, where children are expected to provide material support to parents and where this association is theorized to be a driver of high fertility, there has been limited research examining the subjective experience of parenthood. In this analysis, we analyzed the association between parenthood and life satisfaction for a sample of mid-life women in rural southern Mozambique. Extending previous theoretical and empirical research, we distinguished between children of different ages and children living within and away from the parental home. Results show that having young children living in the home is negatively associated with life satisfaction, while having older children living away from the home is positively associated with life satisfaction, particularly if those children are living outside the country.

Having children under age 6 is associated with lower levels of life satisfaction for mid-life women in our setting in rural southern Mozambique. This association is robust to different specifications and is not fully mediated by measures of physical health, mental health, or economic conditions. This finding is consistent with results from other studies in high-fertility contexts in Africa (Clark et al., 2020; Conzo et al., 2017), as well as some studies in low-fertility contexts (Myrskylä & Margolis, 2014). Some studies in higher-income settings have proposed that the negative impact of children on parental well-being is related to the challenges of combining parenthood with paid work (Glass et al., 2016). The consistency of this negative association even in low-income settings where few women are in the formal labor market points to the inherent demands of parenting young children in addition to possible institutional constraints.

Theories of fertility in sub-Saharan Africa propose that birth rates are high in the region because children make substantial contributions to farming and household production (Shapiro & Gebreselassie, 2013). However, our results show that, at least in this context, the largest benefits to subjective well-being are not from adolescent and young adult children living in the home, who are most likely to contribute to household labor. Rather, children living outside the home, and especially outside the country, are most associated with women’s life satisfaction. Although we do not have direct measures of these children’s activities, given the nature of migration in this context, it is likely that they are gainfully employed and able to contribute financially to their parental household, and women may anticipate future financial support. The association for older children living outside of the country is significantly mediated by household economic conditions, lending support to this potential explanation. In addition, having children living outside the parental home may change the nature of the parent–child relationship in ways that are beneficial for women. More generally, moving out of the parental home, and especially to a higher-income yet geographically proximate foreign setting such as RSA, may be seen as evidence of a successful transition to adulthood, or women may gain social status from having a child living away from home. Studies in other rural, agricultural contexts on how children’s migration is associated with parental outcomes have found mixed results, but these studies are typically focused on older parents (Kuhn et al., 2011; Song, 2017; Thapa et al., 2024); future research could explore in more depth the reasons for this association with the well-being of middle-aged parents. Our findings also suggest that policies aimed at enhancing young people’s successful transition to adulthood may indirectly impact their middle-aged mothers’ well-being.

As noted earlier, our analysis did not differentiate between sons and daughters, based on exploratory analyses showing few statistically significant differences (Appendix 2). However, the transition to adulthood is strongly gendered in this context. For example, marriage is the most salient transition to adulthood for girls, and girls most often leave the household after marriage. Boys typically begin working for pay before marriage and may leave the household to look for work. Some boys, especially youngest sons who traditionally inherit the parental home, may stay in (or return to) the household after marriage and bring their wives into the household. Thus, the implication of coresidence likely differs for boys and girls, and statistical controls may not fully account for the complex dependencies between age, gender, and coresidence in the relationship between children’s characteristics and mothers’ subjective well-being.

We used cross-sectional models to describe associations, including mediation, and did not attempt to formally estimate causal relationships. Previous research in similar settings has found that associations between life satisfaction and parenthood persist when incorporating strategies to account for selection and confounding, such as instrumental variables or lagged dependent variables (Clark et al., 2020; Conzo et al., 2017). Still, some of the associations described here may be partly attributable to reverse causation, and results should be interpreted as descriptive of overall life course trajectories rather than causal.

Our data come from a study of middle-aged women who were living mainly in rural areas of four districts of Gaza Province, Mozambique, and reflect the experiences of these women and their specific social context. These findings may be particularly relevant for policy makers seeking to understand and improve women’s well-being in Mozambique. In addition, the setting for our study shares some salient characteristics with other rural agricultural settings, particularly other settings in sub-Saharan Africa, such as a dependence on subsistence agriculture and increasing vulnerability to climate change; limited local labor markets; high levels of labor out-migration; reliance on family support rather than formal social safety net programs; and limited opportunities for women outside of family roles. We expect that our findings also apply to other settings that share these characteristics, but future research should be carried out in other contexts and with a comparative lens to understand how specific elements of social context shape the experience of parenthood and returns to childbearing.

Our findings highlight the need for more scholarly attention to the range of ways that children may (or may not) contribute to parental well-being, including subjective well-being. Growing economic instability, accelerating climate change, and rising labor migration have profound implications for household livelihood strategies in rural economies. These transformations also shape children’s transitions to adulthood, by extending schooling, delaying marriage and childbearing, and shifting economic opportunities. Our results point to the potential intergenerational consequences of young people’s experiences in adolescence and early adulthood. Classic perspectives on high fertility that link the value of children to their household and agricultural labor need to be adjusted to account for changes in intergenerational relationships across the life course.

In seeking to understand continued high fertility, it is also important to consider how the long-term implications of childbearing are perceived and understood by people making decisions about having children. It is possible that social and economic conditions are changing more rapidly than social expectations about parenting and intergenerational support. If children’s capacity to provide support to parents does not match parental expectations, this mismatch could have implications both for parental material well-being and for the emotional dimensions of the relationship between parents and adult children. In these conditions of rapid social transformation, it is important to consider the social and emotional aspects of parenthood and family relationships as well as economic exchange.

## Supplementary Information

Below is the link to the electronic supplementary material.

Supplementary Material 1

## Footnotes

1 Family Migration and Early Life Outcomes. J. E. Glick, Program Director. Award Number P01HD080659, Eunice Kennedy Shriver National Instiute of Child Health & Human Development, National Institutes of Health.

2 In a few cases, women reported the same child as both living outside the household and living in the household. For example, migrant sons whose wife and children lived in the household were sometimes reported as both absent and present. These “misreports” reflect the complexity of migrant ties to the household; we treat these counts at face value. Alternative models treating children who had not slept in the household for most nights in the past month as non-resident produce essentially identical results.
