# Supplementary material for: Parenthood and Women’s Subjective Well-being in a Low-income, High-fertility Context: A Case Study from Rural Gaza Province, Mozambique
Source: Popul Res Policy Rev. 2025 Oct 31;44(6):57. doi: 10.1007/s11113-025-09978-8 (PMC12578741; doi:10.1007/s11113-025-09978-8)
Supplement: Supplementary file 1 — Supplementary Material 1 [file 11113_2025_9978_MOESM1_ESM.docx]

Parenthood and women’s subjective well-being in a low-income, high-fertility context: A case study from rural Gaza Province, Mozambique

**Appendix 1: Text of questions on food security**

Speaking of food consumption, in the past six months, more or less how many times did the following happen?

A. There was a shortage of food in your household?

B. There was little variety of food for children in your household?

C. You were worried that you would not have enough to eat?

D. You ate less than what you thought you should eat?

E. You skipped a meal because of scarcity of food?

F. You spend a whole day without eating anything?

For all questions, response categories are “many times” (1), “sometimes” (2), and “never”

(3). The scale is created by averaging responses across all items.

This scale is adapted from the Household Food Insecurity Access Scale.

Coates, J., Swindale, A., & Bilinsky, P. (2007). Household Food Insecurity Access Scale (HFIAS) for measurement of food access: Indicator guide: version 3.

Appendix 2: Alternative specifications

**Appendix 2: Alternative model specifications**

This appendix includes the following tables:

Table A2.1: ordered logit models and partial proportional odds ordered logit models

Table A2.2: models with each age and residential category tested separately

Table A2.3: models using dichotomous measures of any children in each age and residential category

Table A2.4: models with interactions

Table A2.5: models testing sons and daughters separately

Table A2.1. Ordered logit and partial proportional odds regressions predicting life satisfaction as a function of number and characteristics of children

|  | Ordered logit | | | Partial proportional odds: cut 1 | | | Partial proportional odds: cut 2 | | | Partial proportional odds: cut 3 | | |
| --- | --- | --- | --- | --- | --- | --- | --- | --- | --- | --- | --- | --- |
|  | b | se |  | b | se |  | b | se |  | b | se |  |
| *Number and characteristics of children* | -0.21 | 0.06 | ** | -0.33 | 0.14 | * | -0.24 | 0.07 | *** | -0.11 | 0.09 |  |
| Children 0-5 in the household | -0.06 | 0.04 |  | 0.03 | 0.09 |  | -0.02 | 0.04 |  | -0.17 | 0.06 | ** |
| Children 6-14 in the household | 0.05 | 0.05 |  | 0.23 | 0.10 | * | 0.04 | 0.05 |  | -0.01 | 0.06 |  |
| Children 15+ in the household |  |  |  |  |  |  |  |  |  |  |  |  |
| Children living outside the household in Mozambique | 0.08 | 0.06 |  | -0.08 | 0.12 |  | 0.01 | 0.07 |  | 0.29 | 0.08 | *** |
| Children living outside Mozambique | 0.40 | 0.15 | ** | 0.21 | 0.39 |  | 0.19 | 0.16 |  | 0.63 | 0.17 | *** |
| *Controls* |  |  |  |  |  |  |  |  |  |  |  |  |
| Child deaths | -0.01 | 0.04 |  | -0.01 | 0.04 |  | -0.01 | 0.04 |  | -0.01 | 0.04 |  |
| Age | -0.13 | 0.08 |  | -0.14 | 0.08 |  | -0.14 | 0.08 |  | -0.14 | 0.08 |  |
| Age squared | 0.001 | 0.001 |  | 0.001 | 0.001 |  | 0.001 | 0.001 |  | 0.001 | 0.001 |  |
| Marital status (reference = married to non-migrant) |  |  |  |  |  |  |  |  |  |  |  |  |
| Unmarried | -0.97 | 0.12 | *** | -0.97 | 0.12 | *** | -0.97 | 0.12 | *** | -0.97 | 0.12 | *** |
| Married to a “successful” migrant | 0.53 | 0.11 | *** | 0.54 | 0.11 | *** | 0.54 | 0.11 | *** | 0.54 | 0.11 | *** |
| Married to an “unsuccessful” migrant | -0.43 | 0.16 | ** | -0.43 | 0.16 | ** | -0.43 | 0.16 | ** | -0.43 | 0.16 | ** |
|  |  |  |  |  |  |  |  |  |  |  |  |  |
| Intercept | -6.36 | 1.59 | *** | 6.28 | 1.59 | *** | 3.8 | 1.58 | * | 1.81 | 1.58 |  |
|  | -3.71 | 1.58 | * |  |  |  |  |  |  |  |  |  |
|  | -1.64 | 1.58 |  |  |  |  |  |  |  |  |  |  |

Data: Men’s Migrations and Women’s Lives project. N=1887 women interviewed at W5, missing values on independent variables imputed using multiple imputation with chained equations. In partial proportional odds model, proportional odds assumptions are relaxed for variables measuring the number and characteristics of children (based on Brant test from ordered logit model). *: 0<.05; **: p<.01; ***: p<001.

Table A2.2: OLS regression predicting life satisfaction as a function of age and characteristics of children, age and residential categories modeled independently

|  | Model 1 | | | | Model 2 | | | | Model 3 | | |
| --- | --- | --- | --- | --- | --- | --- | --- | --- | --- | --- | --- |
|  | b | se |  | b | | se |  | b | | se |  |
| *Number and characteristics of children* |  |  |  |  | |  |  |  | |  |  |
| Children 0-5 in the household | -0.09 | 0.03 | *** |  | |  |  |  | |  |  |
| Children 6-14 in the household |  |  |  | -0.04 | | 0.02 | * |  | |  |  |
| Children 15+ in the household |  |  |  |  | |  |  | 0.02 | | 0.02 |  |
| *Controls* |  |  |  |  | |  |  |  | |  |  |
| Child deaths | -0.002 | 0.02 |  | -0.01 | | 0.02 |  | 0.001 | | 0.02 |  |
| Age | -0.06 | 0.03 |  | -0.04 | | 0.03 |  | -0.06 | | 0.03 |  |
| Age squared | 0.001 | 0.0004 |  | 0.001 | | 0.0004 |  | 0.001 | | 0.0004 |  |
| Marital status (reference = married to non-migrant) |  |  |  |  | |  |  |  | |  |  |
| Unmarried | -0.38 | 0.05 | *** | -0.37 | | 0.05 | *** | -0.35 | | 0.05 | *** |
| Married to a “successful” migrant | 0.22 | 0.04 | *** | 0.22 | | 0.05 | *** | 0.23 | | 0.05 | *** |
| Married to an “unsuccessful” migrant | -0.18 | 0.06 | ** | -0.18 | | 0.06 | ** | -0.17 | | 0.06 | ** |
|  |  |  |  |  | |  |  |  | |  |  |
| Intercept | 3.98 | 0.64 | *** | 3.63 | | 0.64 | *** | 3.83 | | 0.66 | *** |

|  | Model 4 | | | Model 5 | | |
| --- | --- | --- | --- | --- | --- | --- |
|  | b | se |  | b | se |  |
| *Number and characteristics of children* |  |  |  |  |  |  |
| Children living outside the household in Mozambique | 0.03 | 0.03 |  |  |  |  |
| Children living outside Mozambique |  |  |  | 0.15 | 0.06 | * |
| *Controls* |  |  |  |  |  |  |
| Child deaths | 0.00002 | 0.02 |  | -0.0001 | 0.02 |  |
| Age | -0.05 | 0.03 |  | -0.04 | 0.03 |  |
| Age squared | 0.001 | 0.0004 |  | 0.001 | 0.0004 |  |
| Marital status (reference = married to non-migrant) |  |  |  |  |  |  |
| Unmarried | -0.35 | 0.05 | *** | -0.35 | 0.05 | *** |
| Married to a “successful” migrant | 0.23 | 0.05 | *** | 0.23 | 0.05 | *** |
| Married to an “unsuccessful” migrant | -0.17 | 0.06 | ** | -0.17 | 0.06 | ** |
|  |  |  |  |  |  |  |
| Intercept | 3.67 | 0.64 | *** | 3.61 | 0.64 |  |

Data: Men’s Migrations and Women’s Lives project. N=1887 women interviewed at W5, missing values on independent variables imputed using multiple imputation with chained equations. *: 0<.05; **: p<.01; ***: p<001.

Table A2.3: OLS regression predicting life satisfaction as a function of having children in specified age and residence categories

|  | b | se |  |
| --- | --- | --- | --- |
| *Number and characteristics of children* |  |  |  |
| Children living in the household |  |  |  |
| Any children 0-5 in the household | -0.14 | 0.04 | *** |
| Any children 6-14 in the household | -0.07 | 0.06 |  |
| Any children 15 and older in the household | 0.01 | 0.05 |  |
| Children 15 and older living outside the household |  |  |  |
| Any children living in Mozambique | 0.01 | 0.05 |  |
| Any children living outside Mozambique | 0.16 | 0.08 | + |
| *Controls* |  |  |  |
| Child deaths | -0.003 | 0.02 |  |
| Age | -0.05 | 0.03 |  |
| Age squared | 0.001 | 0.0004 |  |
| Marital status (reference = married to non-migrant) |  |  |  |
| Unmarried | -0.38 | 0.05 | *** |
| Married to a “successful” migrant | 0.23 | 0.05 | *** |
| Married to an “unsuccessful” migrant | -0.17 | 0.06 | ** |
|  |  |  |  |
| Intercept | 3.89 | 0.69 | *** |

Data: Men’s Migrations and Women’s Lives project. N=1887 women interviewed at W5, missing values on independent variables imputed using multiple imputation with chained equations. +: p<.10; *: 0<.05; **: p<.01; ***: p<001.

Table A2.4: OLS regression predicting life satisfaction as a function of number and characteristics of children, interactions between categories

|  | Model 1 | | | Model 2 | | | |  |
| --- | --- | --- | --- | --- | --- | --- | --- | --- |
|  | b | se |  | | b | se |  | |
| *Number and characteristics of children* |  |  |  | |  |  |  | |
| Children living in the household |  |  |  | |  |  |  | |
| Children 0-5 in the household | -0.15 | 0.05 | ** | | -0.09 | 0.03 | *** | |
| Children 6-14 in the household | -0.05 | 0.02 | * | | -0.02 | 0.02 |  | |
| Children 15 and older in the household | 0.02 | 0.02 |  | | 0.03 | 0.03 |  | |
| Children 15 and older living outside the household |  |  |  | |  |  |  | |
| Children living in Mozambique | 0.04 | 0.03 |  | | 0.04 | 0.03 |  | |
| Children living outside Mozambique | 0.16 | 0.06 | ** | | 0.15 | 0.06 | ** | |
| Interactions |  |  |  | |  |  |  | |
| Children 6-14 x children 0-5 | 0.03 | 0.02 |  | |  |  |  | |
| Children 15 and older x children 0-5 |  |  |  | | -0.01 | 0.01 |  | |
| *Controls* |  |  |  | |  |  |  | |
| Child deaths | -0.0006 | 0.02 |  | | -0.003 | 0.02 |  | |
| Age | -0.05 | 0.03 |  | | -0.05 | 0.03 |  | |
| Age squared | 0.001 | 0.0004 |  | | 0.0005 | 0.0004 |  | |
| Marital status (reference = married to non-migrant) |  |  |  | |  |  |  | |
| Unmarried | -0.38 | 0.05 | *** | | -0.38 | 0.05 | *** | |
| Married to a “successful” migrant | 0.23 | 0.04 | *** | | 0.23 | 0.05 | *** | |
| Married to an “unsuccessful” migrant | -0.18 | 0.06 | ** | | -0.18 | 0.06 | ** | |
|  |  |  |  | |  |  |  | |
| Intercept | 4.05 | 0.66 | *** | | 3.98 | 0.66 | *** | |

Data: Men’s Migrations and Women’s Lives project. N=1887 women interviewed at W5, missing values on independent variables imputed using multiple imputation with chained equations. *: 0<.05; **: p<.01; ***: p<001.

Table A2.5: OLS regression predicting life satisfaction as a function of number and characteristics of children and by child gender

|  | b | se |  |
| --- | --- | --- | --- |
| *Number and characteristics of children* |  |  |  |
| Children living in the household |  |  |  |
| Children 0-5 in the household | -0.09 | 0.03 | *** |
| Boys 6-14 in the household | -0.04 | 0.02 | * |
| Girls 6-14 in the household | -0.02 | 0.02 |  |
| Boys 15 and older in the household | 0.01 | 0.02 |  |
| Girls 15 and older in the household | 0.04 | 0.03 |  |
| Children 15 and older living outside the household |  |  |  |
| Boys living in Mozambique | -0.09 | 0.05 |  |
| Girls living in Mozambique | 0.07 | 0.03 | * |
| Boys living outside Mozambique | 0.15 | 0.07 | * |
| Girls living outside Mozambique | 0.19 | 0.14 |  |
| *Controls* |  |  |  |
| Child deaths | -0.001 | 0.02 |  |
| Age | -0.05 | 0.03 |  |
| Age squared | 0.001 | 0.0004 |  |
| Marital status (reference = married to non-migrant) |  |  |  |
| Unmarried | -0.38 | 0.05 | *** |
| Married to a “successful” migrant | 0.23 | 0.04 | *** |
| Married to an “unsuccessful” migrant | -0.18 | 0.06 | ** |
|  |  |  |  |
| Intercept | 4.08 | 0.66 | *** |

Data: Men’s Migrations and Women’s Lives project. N=1887 women interviewed at W5, missing values on independent variables imputed using multiple imputation with chained equations. *: 0<.05; **: p<.01; ***: p<001.
